# Depression prediction based on LassoNet-RNN model: A longitudinal study

**DOI:** 10.1016/j.heliyon.2023.e20684

**Published:** 2023-10-05

**Authors:** Jiatong Han, Hao Li, Han Lin, Pingping Wu, Shidan Wang, Juan Tu, Jing Lu

**Affiliations:** a School of Computer Science, Nanjing Audit University, China; b Jiangsu Key Laboratory of Public Project Audit, School of Engineering Audit, Nanjing Audit University, China; c Key Laboratory of Modern Acoustics (MOE), School of Physics, Nanjing University, China

**Keywords:** Depression, LassoNet-RNN, CHARLS, Characteristic variables

## Abstract

Depression has become a widespread health concern today. Understanding the influencing factors can promote human mental health as well as provide a basis for exploring preventive measures. Combining LassoNet with recurrent neural network (RNN), this study constructed a screening model ,LassoNet-RNN, for identifying influencing factors of individual depression. Based on multi-wave surveys of China Health and Retirement Longitudinal Study (CHARLS) dataset (11,661 observations), we analyzed the multivariate time series data and recognized 27 characteristic variables selected from four perspectives: demographics, health-related risk factors, household economic status, and living environment. Additionally, the importance rankings of the characteristic variables were obtained. These results offered insightful recommendations for theoretical developments and practical decision making in public health.

## 1 Introduction

Depression has attracted mounting attention due to its high prevalence worldwide. Globally, nearly 350 million people suffer from depression [1]. Moreover, since the outbreak of the COVID-19 epidemic, there has been a 25 % increase in the rates of depression and anxiety worldwide [2,3]. Depression often co-occurs with other multifactorial diseases, such as diabetes, cancer, heart disease, and dementia. It can complicate and prolong the course of these illnesses, placing a significant burden on families and society [4]. According to the World Health Organization (2020), the global prevalence of depression reaches 5 % in all countries, and the prevalence in China is higher than the world average, with a clear upward trend in recent years. Depression is a typical mood disorder characterized by significant and persistent low mood and high rates of suicide, which causes profound suffering to individuals and families and impairs social functioning and economic productivity [[5], [6], [7], [8]]. In addition, the risk of developing depression peaks in middle and old age [9,10], where the psychological effects of depression lead to the deterioration of all physical functions and have long-term negative impacts on the psychological and social functioning of the individual. Given the non-trivial effect of depression on human beings and society, it is vital to identify the key factors that influence people's depressive tendencies and to take appropriate measures against the salient determinants of depression.

The study of the pathogenesis, detection, and influencing elements of depression is an open question [11]. There are many factors that influence depressive tendencies and their prognosis, including genetics, diet, and hormone levels [12]. Recent studies are increasingly revealing the features that contribute to depression, and feature identification methods are constantly evolving. Traditional statistical models generally use a single predictor with poor predictive accuracy, while multiple linear regression models use multiple predictors to identify key factors that influence a person's propensity for depression, and they have achieved good results. However, the number of predictors must be relatively smaller than the sample size to ensure the stability and reproducibility of traditional linear models. Additionally, conventional statistical regression models are very susceptible to bias and are substantially impacted by original bias in the data sample. Due to these limitations, traditional multiple regression is unable to thoroughly analyze the sample data and utilize the available variables effectively [13,14]. Thus, Machine learning is increasingly being used to detect and aid in the diagnosis of depression because of its potent data processing, mining skills and capacity to discover patterns in data from a large number of samples without being subject to bias [13,[15], [16], [17]]. And it is feasible for machine learning methods to iterate and simultaneously analyze non-linear, high-dimensional correlations between risk factors and capture the temporal relationships between risk factors [18]. There has been extensive research on depression using machine learning techniques [19,20]. For example, convolutional neural networks (CNNs), deep belief networks, and recurrent neural networks (RNNs) are representative machine learning techniques that are applied to study the pathogenesis and influences of depression [21,22]. In a trial of sequencing treatment alternatives for the relief of depression [23], logistic regression models were good predictors of treatment-resistant depression [24]. Similarly, Long Short Term Memory (LSTM) models predict well the co-occurrence of diabetes and depression [25], which have been widely used in healthcare interdisciplinary clinical trials to predict moderated cure effects for depression [26]. Ensemble model from random forests and elasticity networks can be used to analyze the influences of self-directed intervention-based depression in patients [27,28]. In addition, machine learning techniques can be applied to assist in the diagnosis of depression based on sociodemographic characteristics, comorbidity data [[29], [30], [31], [32]] or brain imaging data [33], to screen for risk of depression and predict the risk of future depressive episodes [29,34].

LassoNet is an effective model for feature selection [35]. It is a generalization of the Lasso linear model and extends the feature sparsity of Lasso regression to feedforward neural networks [36]. The LassoNet feature selection model can be used to identify factors driving depression. However, because of the continuity of the characteristics of the factors influencing depressive tendencies, cross-sectional data cannot contain significant continuous characteristics. A time series representation better reflects the true nature of the features, and the LassoNet model is limited in capturing features based on temporal relationships [37]. However, due to LassoNet's inherent limitation in handling time-series data [38], we propose a new model in this paper to address this issue.

Our main aim is to integrate LassoNet with RNN to construct the LassoNet-RNN selection model to obtain an importance ranking of factors affecting an individual's depression by analyzing multivariate time-series data, identifying, and recognizing 27 characteristic variables from four dimensions: demographics, health-related risk factors, family economic status and living environment. The ultimate aim is to provide useful theoretical developments and recommendations that can inform and guide practical decision-making in the field of public health. The main contributions of this paper are as follows. (1) This study uses the LassoNet-RNN model to identify important characteristics that influence people's depressive tendencies with the CHARLS dataset, and to identify important factors that influence depressive tendencies in mental health care. (2) This study constructs a new feature selection model, LassoNet-RNN, which can be used in depression studies. (3) This study will help healthcare professionals identify people with depressive tendencies and raise awareness of depression in society, promoting overall mental health.

## 2 Materials and methods

### 2.1 China Health and Retirement Longitudinal Study (CHARLS)

CHARLS collects high-quality data on households and individuals from 150 counties and 450 communities (villages) in 28 provinces in China to promote interdisciplinary research. The national baseline survey of the CHARLS database was conducted in 2011, followed by the return surveys in 2013, 2015, and 2018 respectively. The CHARLS sample was designed to ensure an unbiased and representative sample [39]. Each survey interviewee signed an informed consent form before participating in the survey. Detailed descriptions of the CHARLS dataset and the full data are available on its official website. Data is collected by trained investigators through face-to-face interviews [40]. The CHARLS survey covers demographic background, health status and functioning, cognition and depression, health care and insurance, work and retirement information [41], pensions, household assets, and property and debt status. Due to excessive missing data from the 2011 baseline survey, this study is based on the 2013, 2015, and 2018 data from the CHARLS. Table 1 describes the age and gender composition of the CHARLS sample.

**Table 1:** Basic information on CHARLS database.

Age	2013			2015			2018		
	Sum	Male	Female	Sum	Male	Female	Sum	Male	Female
≤44	435	76	359	718	137	581	255	31	224
45–49	3153	1398	1755	3175	1503	1672	1960	820	1140
50–54	2827	1348	1479	3551	1694	1857	3500	1664	1836
55–59	3406	1655	1751	3095	1532	1563	3045	1429	1616
60–64	3152	1581	1571	3594	1723	1871	3375	1665	1710
65–69	2084	1051	1033	2537	1297	1240	3162	1512	1650
70–74	1466	756	710	1679	823	856	1996	1002	994
75–79	981	511	470	1083	577	506	1330	656	674
≥80	750	331	419	841	368	473	1193	561	632
Number of people	18,254	8707	9547	20,273	9654	10,619	19,816	9340	10,476

Note: 10 in 2013 and 11 in 2015 lack age information.

### 2.2 Study variables

#### 2.2.1 Label variables

Depressive tendencies were the label variable for this study. The Center for Epidemiologic Studies Short Depression Scale (CESD-10) was provided in the CHARLS dataset to measure depression scores. The CESD-10 involves 10 questions about the respondent's feelings and behaviors, as shown in Table 2, and respondents were asked to select the frequency of the event from four options. Of these 10 questions, questions 5 and 8 were positive, so respondents were given a score of 3 when they selected 'Rarely or none of the time', decreasing in order; the remaining 8 questions were given a score of 0 when respondents selected 'Rarely or none of the time', increasing in order. The score values represented by these options were added together to obtain a depressive disposition score ranging from 0 to 30. The CESD-10 has been used with high validity in the general population and has adequate reliability and validity in studies of depressive disposition in China, especially among older adults [42]. The higher the score, the greater the risk of developing depression. In this study, individuals with CESD-10 scores above 10 are labeled as the depressive group and those with scores less than or equal to 10 are labeled as the normal group [43].

**Table 2:** CESD-10 depression scale questions and answers.

	Questions	Answers
1	I was bothered by things that don't usually bother me.	1. Rarely or none of the time (<1 day) 2. Some or a little of the time (1–2 days) 3. Occasionally or a moderate amount of the time (3–4 days) 4. Most or all of the time (5–7 days)
2	I had trouble keeping my mind on what I was doing.	
3	I felt depressed.	
4	I felt everything I did was an effort.	
5	I felt hopeful about the future.	
6	I felt fearful.	
7	My sleep was restless.	
8	I was happy.	
9	I felt lonely.	
10	I could not get "going".	

#### 2.2.2 Characteristic variables

For the 0–1 characteristic variables such as smoking or not, and whether or not to take a lunch break, the value of "1″ is assigned when the feature value is "yes" and "0″ is assigned when the feature value is "no". For chronic disease status, the presence or absence of chronic diseases such as hypertension, dyslipidemia and diabetes are standardized as a characteristic variable for the presence or absence of chronic diseases. Variables such as gender and place of residence are also defined as 0–1 variables in this study as they have two responses. In addition, the higher the education value, the higher the level of education. Intergenerational financial support includes whether children and parents have financial interactions, including whether parents pay monthly living expenses to their children and whether children pay monthly pensions to their parents. Social participation is a portrayal of an individual's social participation and consists of 10 activities, such as going out or chatting with friends and going to school or attending any training courses. Respondents were asked to recall whether they had done any of the above things in the past month, and social participation was given a value size of 0–10, indicating the number of those 10 activities done. There are three values for drinking frequency, representing never drinking (0), drinking less than once a month (1), and drinking more than once a month (2). The amount spent in a year includes clothing consumption, the cost of heating for the household, the cost of expensive consumer goods and appliances, house rent, etc. The specific relevant characteristic variables are defined as shown in Table 3.

**Table 3:** Definition of relevant characteristic variables.

Characteristic variables	Definition
Gender	0: Female; 1: Male
Age	45 years and above
Education	Values range from 0 to 9
Whether living with a spouse	0: No; 1: Yes
Residence	0: Rural; 1: Urban
Intergenerational financial support	Whether there is financial contact with parents or children (0: No; 1: Yes)
Availability of health insurance	0: No; 1: Yes
Social participation	Includes 10 activities such as stringing, playing mahjong, dancing, doing charity, etc., taking values from 0 to 10
Sleeping time at night	Length of night sleep (in hours)
Lunch break or not	0: No; 1: Yes
Recently hospitalized	0: No; 1: Yes
Drinking frequency	Frequency of drinking includes nothing, less than once a month and more than once a month, with values ranging from 0 to 2
Smoking or not	0: No; 1: Yes
Presence of chronic diseases	Chronic conditions include: hypertension, dyslipidemia, diabetes, cancer or malignancy, lung disease, liver disease, heart disease, stroke, kidney disease, mental illness, arthritis and asthma. (0: No; 1: Yes)
Availability of pensions	0: No; 1: Yes
Whether working in agriculture	0: No; 1: Yes
Whether self-employed or privately owned	0: No; 1: Yes
Retirement	0: No; 1: Yes
Amount spent in a year	Amount of household consumption in a year
Whether owe a debt	0: No; 1: Yes
Availability of piped water	0: No; 1: Yes
Availability of bathing facilities	0: No; 1: Yes
Availability of gas facilities	0: No; 1: Yes
Availability of heating	0: No; 1: Yes
Availability of broadband facilities	0: No; 1: Yes
Home tidiness	Values range from 0 to 4
Indoor temperature	Values range from 0 to 4

### 2.3 Model building

#### 2.3.1 Multivariate time series

Time series data is a series of real-valued points of data measured continuously over time for the purpose of describing the behavior of a system [44]. Time series can be divided into univariate and multivariate time series. Multivariate time series data is a collection of time series describing different aspects of a particular time phenomenon [45]. In a departure from traditional classification problems, time series classification emphasizes the temporal relationships between attributes. Time series have many real-world applications, such as healthcare, human activity recognition [46], stock prediction [47], speech recognition [48], and many more. In fact, any classification problem, using data recorded considering some notion of order, can be mapped as a multivariate time series classification problem [49].

In many cases, multivariate time series data are characterized by high dimensionality and spatiotemporal dependence, which makes it difficult to model them effectively by classical statistical methods [50]. The central complexity of multivariate time series classification is that discriminatory features may exist in the interactions between dimensions, rather than just autocorrelation in individual series. In multivariate time series classification, features may or may not depend on both dimensions [51]. Since the dataset consists of multivariate time series, a time series dataset can be defined as a tensor of shape (N, Q, M) [52], where N is the number of samples in the dataset, Q is the maximum number of time steps of all variables in it, and M is the number of variables processed at each time step.

#### 2.3.2 RNN

RNNs are a powerful class of neural network models for processing and predicting sequential data [52,53]. The human brain is an organ with a strongly repetitive recursive structure, and a large number of repetitive recurrences indicate that self-feedback plays an important role in perceiving, acting, learning, and sustaining life. The structural design of RNNs is based on a recursive structure similar to that of the human brain, using a recurrent structure to process time-series data, which overcomes many of the constraints on input and output data that are traditionally associated with machine learning methods [54]. In contrast to feedforward neural networks, there is at least one feedback connection in the RNN and the connections between the nodes of the RNN form a loop along the time series, which helps it to exhibit time-dynamic behavior [55]. The input data of RNN is a variable-length sequence $x=\left(x_{1},\ldots ,x_{T}\right)$ [56]. The sequence to be processed is usually a time sequence, and the evolutionary direction of the sequence is called a time-step. For a time-step t, the loop unit of the RNN can be defined as Equation (1)

(1) $$h_{\left\langle t\right\rangle }=f\left(h_{\left\langle t-1\right\rangle },x_{t}\right)$$

where h is the system state of the RNN, f is an activation function or an encapsulated feedforward neural network [56].

#### 2.3.3 LassoNet model

LassoNet is a neural network framework with global feature selection [35], derives its core idea from the linear Lasso regression model, a generalization of the Lasso linear model. LassoNet extends Lasso regression and its feature sparsity to feedforward neural networks [57]. Thus, LassoNet uses the residuals of the input and output, and the linear and non-linear components of the LassoNet model are jointly optimized to allow the capture of arbitrary non-linear features, extending the use of the Lasso linear model [36]. The method achieves feature sparsity by allowing a feature to participate in the hidden unit only if its linear representation is active. Unlike other feature selection methods for neural networks, LassoNet adopts a modified objective function with constraints to combine feature selection directly with parameter learning. Thus, LassoNet provides an entire regularization path for solutions with a certain degree of feature sparsity. The LassoNet method adopts projective approximate gradient descent and can be directly generalized to deep networks. The structure of LassoNet consists of a single residual connection and an arbitrary feedforward neural network. The residual and hidden layers are jointly passed through a layered soft threshold optimizer.

Equation (2) represent the objective function and constraints of the LassoNet model [35]. The objective function is a representation of the Lasso linear model combined with the L1 regular term, and the constraints indicate that the weight parameter W of each feature on each neuron on the feedforward neural network needs to be less than or equal to M times the parameter of that feature on the linear model [35].

$${minimize}_{\left(\theta ,W\right)}\;L\left(\theta ,W\right)+\lambda {\left\|\theta \right\|}_{1}$$

(2) $$subject\;to\;{\left\|W_{j}^{\left(1\right)}\right\|}_{\infty }\leq M\left|\theta_{j}\right|,j=1,\cdots ,d$$

L(θ,W) in Equation (2) is the residual neural network loss function, θ is the linear part parameter, W is the residual network coefficient, $W_{j}^{\left(1\right)}$ represents the j th component of the first layer coefficient of the residual network, M is the layer multiplier, and λ is the linear part penalty term coefficient. LassoNet does not just sparse the network, but selects relevant input features in a structured way [35].

(3) $$\left|W_{jk}^{\left(1\right)}\right|\leq M\cdot \left|\theta_{j}\right|,k=1,\cdots ,K$$

In the LassoNet framework, feature sparsity is made possible by a controlled trade-off between linear and non-linear components. From Equation (3), when M = 0, $W_{j}^{\left(1\right)}$ is constant equal to 0 and LassoNet is equivalent to Lasso regression. When M = +∞, the model does not impose any constraint on $W_{j}^{\left(1\right)}$ and LassoNet is equivalent to an ordinary feedforward neural network [35].

#### 2.3.4 LassoNet-RNN model

**Fig. 1:**
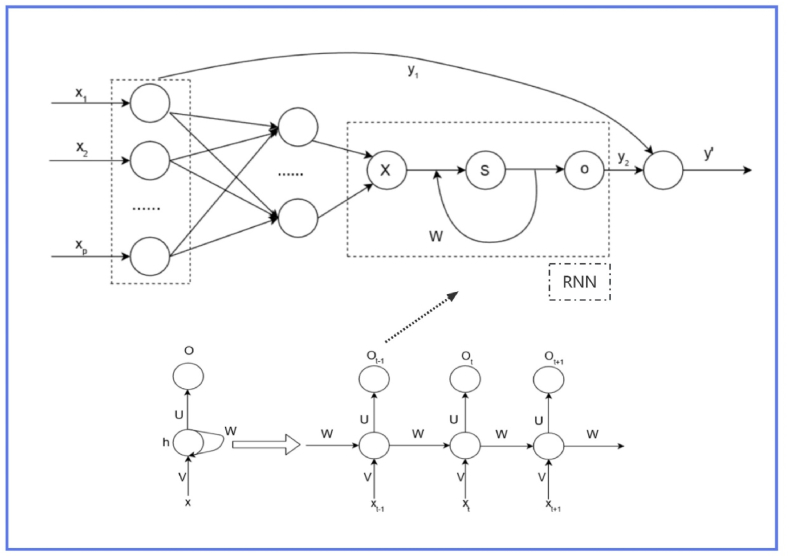
Structure of LassoNet-RNN model.

To enable LassoNet to implement multivariate time series feature selection the standard feedforward neural network in LassoNet needs to be replaced with an RNN capable of recognizing time series, making the model capable of processing sequential data and extending the applicability of the model. It is used to extract important features with a temporal correlation that cannot be captured by a single temporal node. In this study, the hidden layer of LassoNet is replaced by an RNN model, which allows the linear and non-linear models to be jointly optimized to capture arbitrary non-linear and temporal-level features. The diagram of the LassoNet-RNN model is shown in Fig. 1.

The activation function in the LassoNet-RNN model is ReLU. The adaptive moment estimation (Adam) optimizer is a variant of stochastic gradient descent used to update weights of neural networks [58]. Adam is widely used in computer vision, natural language processing, and other practical scenarios. It is characterized by the fact that the learning rate can be adaptively adjusted according to changes in the weights, and it uses the values of the first-order moment estimates and second-order moment estimates of the gradient in order to calculate a learning rate suitable for each parameter change. Thus, the Adam optimizer can calculate adaptive learning rates for different parameters, with efficient computation and fewer memory requirements [37,38]. Lambda_Start is the initial value of the Lambda sequence. The model is trained on Lambda sequence of values until all coefficients are zero.

## 3 Results

### 3.1 Statistical analysis

#### 3.1.1 Factors that influence people's depression

There is no definitive evidence to determine the cause of depression, but many studies suggest that its pathogenesis is closely linked to a variety of factors, including biological, psychological, and social environments. Inter-individual variability in pathophysiological conditions or causes of morbidity may lead to different types of depression. Some individuals with depressive tendencies may present with different types of symptoms at different times. Also, differences in individual exposure to psychosocial stress may lead to different depressive situations. Therefore, more research is needed to gain insight into the factors influencing depression in order to better prevent and alleviate the disorder. There are four broad categories of factors that generally influence an individual's tendency to experience depression: socio-demographic factors [59,60], factors related to one's own health [[61], [62], [63]], economic factors [[64], [65], [66], [67]], and environmental conditions in which one lives [50,[68], [69], [70]]. Such categories are also used in our discussion.

#### 3.1.2 Data pre-processing

**Fig. 2:**
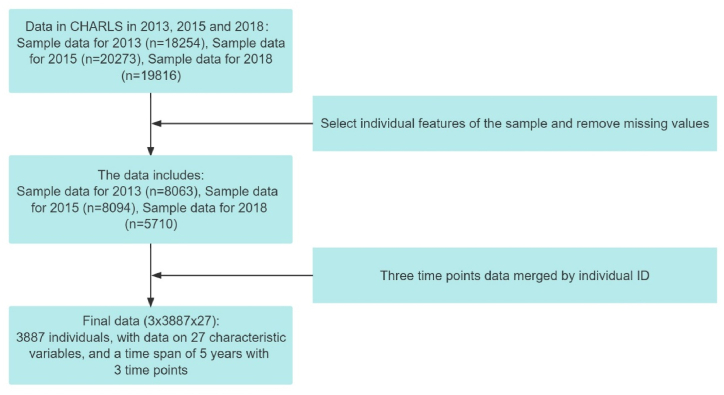
CHARLS data processing flowchart.

Data pre-processing techniques also play an important role in influencing the performance of machine learning models, with pre-processed data improving model accuracy and reducing computational costs during the model learning phase [71]. In this study, the normalization method was chosen to standardize the range, magnitude, format, and type of data, making it easier to carry out subsequent calculations. This study first scaled the multivariate time series data affecting human depression. In the original CHARLS dataset, some variables have problems such as missing feature values and data duplication, which require data pre-processing before building a model for training. Common pre-processing methods for missing values include ignoring, deleting, and interpolating. In terms of interpolation methods, common methods include maximum likelihood estimation, matrix complementation, mean interpolation, and compression perception. In this study, the required features were selected for the sample data of 2013 (n = 18,254), 2015 (n = 20,273), and 2018 (n = 19,816) (n represents the number), and invalid data, non-standardized data, and erroneous data were removed in order to finally obtain the standardized dataset. The pre-processing process for the CHARLS dataset is shown in Fig. 2.

#### 3.1.3 Basic information about depression in the CHARLS dataset study subjects

The number of individuals in the sample used in this study was 3887, with data on a total of 27 characteristic variables spanning a 5-year period with 3-time points (including 2013, 2015, and 2018). The socio-demographic data included 6 variables, including gender, age, education, and whether living with a spouse, among others. The health-related risk factors included 8 variables, including chronic diseases status, social participation, smoking, drinking frequency, and sleeping time at night, among others. The economic status included 5 variables, including amount spent, work status, whether or not they have a pension [72], and debt, among others. The study also included 8 variables on the living environment, including home tidiness, indoor temperature, availability of bathing facilities and availability of gas facilities, among others. In addition, there is a category label for depressive tendencies, indicated by 0 and 1, where 0 means no depressive tendencies and 1 means depressive tendencies.

**Fig. 3:**
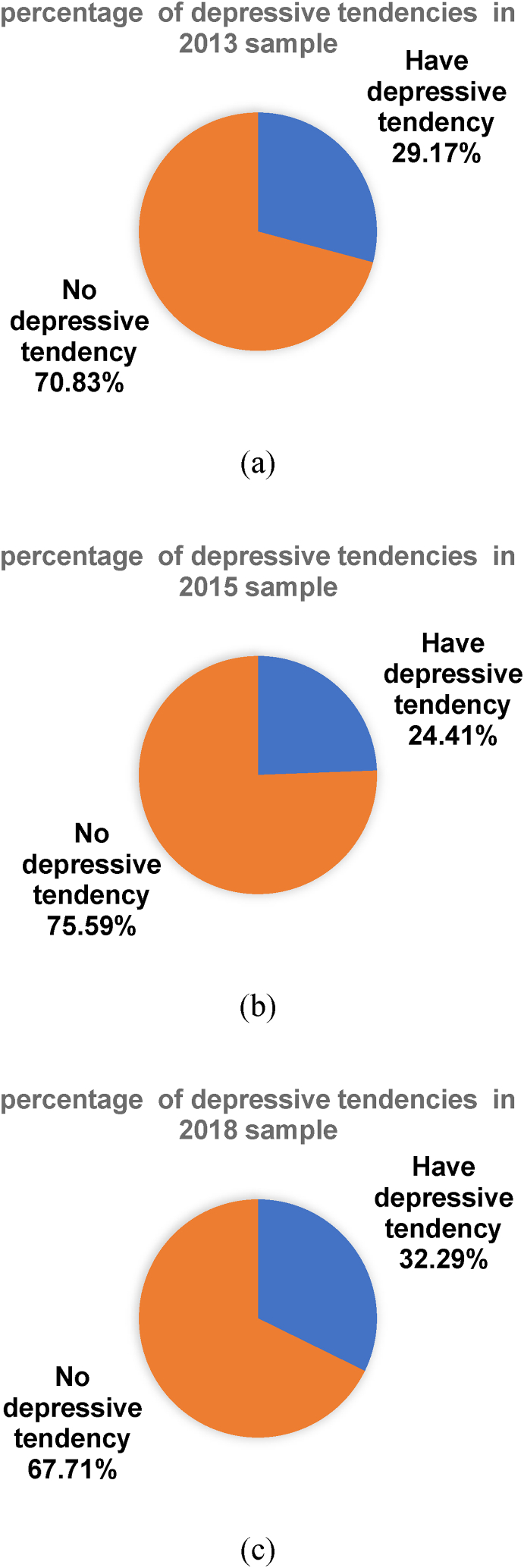
Percentage of depressive tendencies among individuals in sample.

In the 2013 CHARL, a total of 1134 respondents scored over 10 on the CESD-10, representing 29.17 % of the total 2013 data sample. Fig. 3 (a) illustrates the percentage of individuals surveyed in 2013 with CESD-10 scores above 10. In the 2015 CHARL, a total of 949 respondents scored over 10 on the CESD-10, representing 24.41 % of the total 2015 data sample. Fig. 3 (b) illustrates the percentage of individuals surveyed in 2015 with CESD-10 scores above 10. In the 2018 CHARL, a total of 1255 individuals surveyed had CESD-10 scores above 10, representing 32.29 % of the total 2018 data sample, Fig. 3 (c) illustrates the percentage of individuals surveyed in 2018 with CESD-10 scores above 10. Table 4 shows the full characteristics of the sample data used for this experiment.

**Table 4:** Basic information statistics for CHARLS sample data.

		2013			2015			2018		
Characteristics	Group	Average value	Depression	Non-depression	Average value	Depression	Non-depression	Average value	Depression	Non-depression
Gender	Male	0.43	530	1140	0.43	277	1387	0.43	384	1284
	Female		604	1613		672	1551		871	1348
Age	<50	57.46	173	507	59.32	67	221	62.34	24	45
	50–70		894	2066		783	2450		1016	2187
	>70		67	180		99	267		215	400
Residence	Rural	0.28	835	1955	0.28	724	2066	0.28	950	1840
	Urban		299	798		225	872		305	792
Education	<3	2.37	493	1167	2.34	522	1171	2.27	713	1096
	≥3		641	1586		427	1767		542	1536
Whether living with a spouse	Yes	0.87	1006	2394	0.86	773	2583	0.82	982	2223
	No		128	359		176	355		273	409
Intergenerational financial support	Yes	0.70	817	1913	0.89	853	2604	0.86	1104	2229
	No		317	840		96	334		151	403
Availability of health insurance	Yes	0.97	1099	2678	0.75	716	2193	0.97	1213	2564
	No		35	75		233	745		42	68
Social participation	<3	0.93	480	1167	0.90	476	1359	0.84	636	1266
	3–7		394	913		281	837		374	793
	>7		260	673		192	742		245	573
Sleeping time at night		6.25	1134	2753	6.44	949	2938	6.25	1255	2632
Lunch break or not	Yes	0.57	625	1582	0.57	495	1719	0.61	743	1631
	No		509	1171		454	1219		512	1001
Recently hospitalized	Yes	0.08	99	214	0.17	224	447	0.14	235	296
	No		1035	2539		725	2491		1020	2336
Drinking frequency	0	0.62	714	1818	0.61	672	1872	0.58	947	1682
	1		90	210		87	232		72	181
	2		330	725		190	834		236	769
Smoking or not	Yes	0.31	404	812	0.31	234	973	0.29	306	807
	No		730	1941		715	1965		949	1825
Presence of chronic diseases	Yes	0.31	344	869	0.30	328	849	0.49	713	1208
	No		790	1884		621	2089		542	1424
Availability of pensions	Yes	0.80	930	2191	0.85	859	2462	0.90	1131	2381
	No		204	562		90	476		124	251
Whether working in agriculture	Yes	0.26	318	712	0.19	196	542	0.09	89	255
	No		816	2041		753	2396		1166	2377
Whether self-employed or privately owned	Yes	0.10	105	300	0.06	42	187	0.06	119	125
	No		1029	2453		907	2751		1136	2507
Retirement	Yes	0.08	88	214	0.11	116	328	0.21	275	542
	No		1046	2539		833	2610		980	2090
Amount spent in a year		8.02	1134	2753	7.94	949	2938	8.21	1255	2632
Whether owe a debt	Yes	0.06	82	166	0.05	55	155	0.04	60	107
	No		1052	2587		894	2783		1195	2525
Availability of piped water	Yes	0.69	765	1928	0.75	662	2256	0.79	961	2101
	No		369	825		287	682		294	531
Availability of bathing facilities	Yes	0.49	487	1410	0.57	443	1782	0.63	704	1726
	No		647	1343		506	1156		551	906
Availability of gas facilities	Yes	0.09	99	260	0.11	93	342	0.14	146	401
	No		1035	2493		856	2596		1109	2231
Availability of heating	Yes	0.08	82	223	0.08	65	261	0.09	97	261
	No		1052	2530		884	2677		1158	2371
Availability of broadband facilities	Yes	0.21	196	633	0.25	170	799	0.41	421	1157
	No		938	2120		779	2139		834	1475
Home tidiness	0–1	2.00	422	875	2.09	322	848	2.04	493	806
	2		396	995		347	1049		401	892
	3–4		316	883		280	1041		361	934
Indoor temperature	0–1	2.05	48	79	2.06	29	79	2.13	37	54
	2		1000	2446		829	2610		1034	2220
	3–4		86	228		91	249		184	358

#### 3.1.4 Model evaluation

To address the nature of this research problem, this study defines the dichotomous categories of 1 and 0 as positive and negative classes when dealing with labeled data. When using the test dataset to test the model performance, the following four cases are considered: True Positive (TP) is the number of samples that are actually positive and predicted by the model to be positive; False Negative (FN) is the number of samples that are actually positive but predicted by the model to be negative; False Positive (FP) is the number of samples that are actually negative but predicted by the model to be positive; True Negative (TN) is the number of samples that are actually negative and predicted by the model to be negative as well.

Accuracy is a common evaluation metric for classification problems, reflecting the size of the ratio of the number of correctly classified samples to the total number of samples. For traditional data balance classification problems, Accuracy is a good indicator of the performance of the classification algorithm [73]. It is calculated as the ratio of correctly classified data points to the total number of data points [40]. The accuracy rate takes values in the range of 0–1, and the closer the value is to 1, the better the model works. It is calculated as Equation (4).

(4) $$Accuracy=\frac{TP+TN}{TP+FP+FN+TN}$$

The most common method of calculating AUC (Area Under ROC Curve) value is by calculating the area under the ROC (receiver operator characteristic) curve to obtain the AUC value, this method is the most accurate method of calculating AUC, the calculation process is more complicated, the AUC value typically ranges from 0.5 to 1.0. ROC curves and the associated AUC values are powerful tools for evaluating the performance of binary classification models.

The F1-Score is a metric used to evaluate the accuracy of a binary classification model and is widely used in machine learning classification tasks. The F1-Score ranges from 0 to 1, with 1 being the maximum value and 0 being the minimum value [73], which is calculated using Equation (5). To evaluate the performance of a binary classification model, it is usually necessary to evaluate both its accuracy and recall rate. Recall rate is how many of the samples that are actually positive are predicted to be positive. These two metrics are in conflict with each other, and when the accuracy is high, the recall may be low, and vice versa. In practice, researchers need to weigh the trade-offs between accuracy and recall to achieve optimal model performance, depending on the specific needs of the task. While it is possible to obtain high accuracy and recall simultaneously in some simple tasks, this is not always achievable in all tasks. Therefore, the best evaluation metric for each task needs to be chosen on a case-by-case basis.

(5) $$F1-Score=\frac{2*TP}{2*TP+FN+FP}$$

### 3.2 Analysis of human depressive tendencies based on the LassoNet-RNN model

For experiments on the important factors that influence a person's tendency to become depressed, this study randomly divided the pre-processed CHARLS dataset into two groups, with 80 % of the data used in the training set for training and 20 % in the test set for testing, with the training set used to learn and fit the LassoNet-RNN model and the test set used to evaluate the performance of the trained LassoNet-RNN model learned from the training set.

**Fig. 4:**
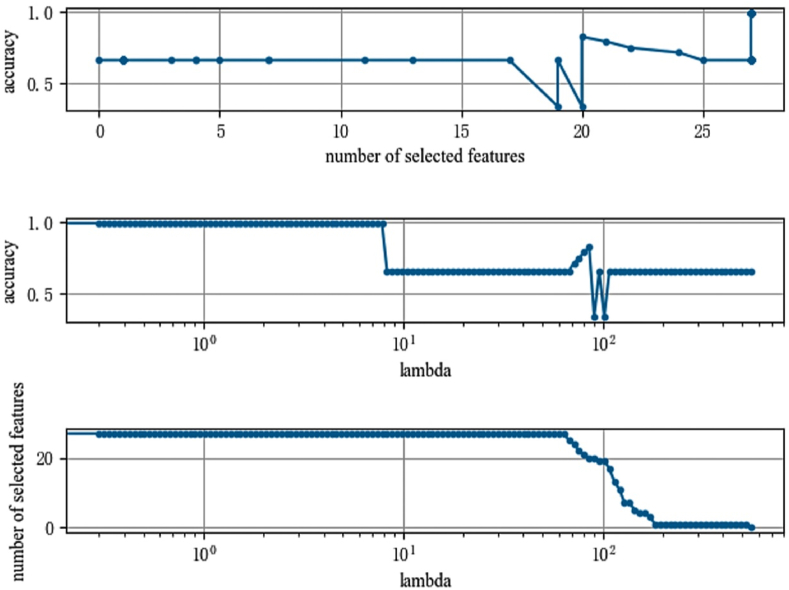
Feature selection diagram (Accuracy).

In this study, the accuracy value is used to evaluate the merit of the training results of the LassoNet-RNN model. The specific experimental results are shown in Fig. 4. From the figure, it can be observed that the accuracy value of the LassoNet-RNN model changes under different numbers of variable selection, the accuracy value does not change much in the early stage when the number of selected characteristic variables is 20, the model achieves a prediction accuracy of about 0.8, furthermore, the accuracy value of LassoNet-RNN reaches higher than 0.99 when the number of variable selection is 27, and when the Lambda value is approximately 1, the model performs best, demonstrating the usefulness of the LassoNet-RNN model on the CHARLS dataset.

**Fig. 5:**
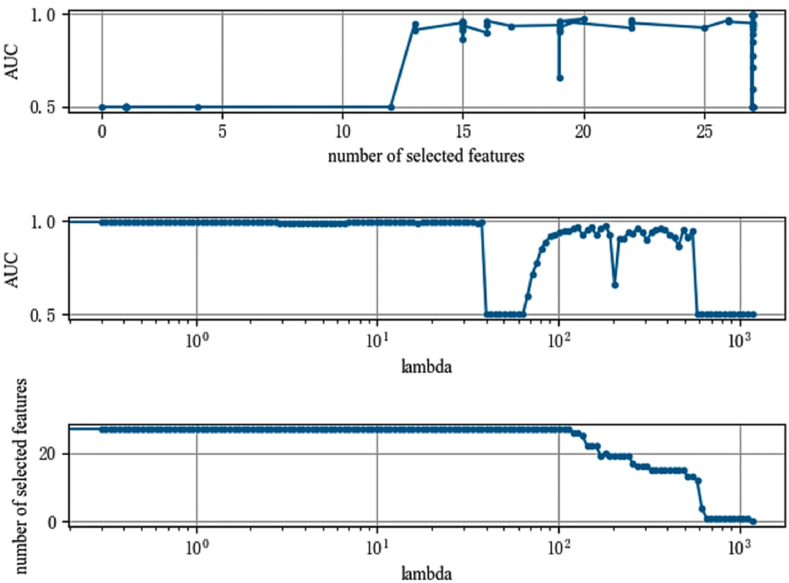
Feature selection diagram (AUC).

**Fig. 6:**
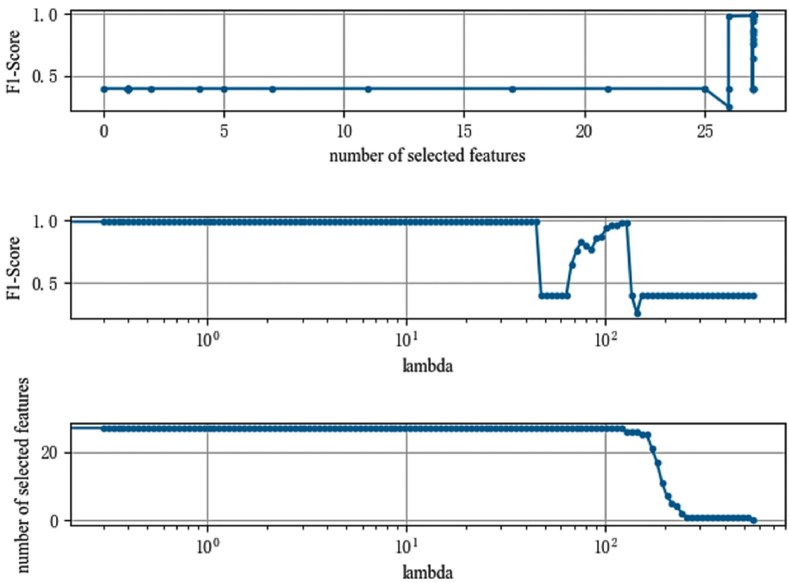
Feature selection diagram (F1-Score).

In order to ensure the robustness of the LassoNet-RNN model on the CHARLS dataset, this study used two metrics, AUC value and F1-Score, as evaluation criteria to select variables again for the data that affect a person's propensity to become depressed, using a similar ratio of training set to test set as when using accuracy as a measure. The results are shown in Fig. 5, Fig. 6. Fig. 5 shows that the AUC value of the model steadily increases as the number of selections increases, and at the point where the selected features reach 15, the model area plateaus and the AUC value approaches 1.0. As shown in Fig. 6, the F1-Score of the LassoNet-RNN model eventually reaches 1.0 on this data, demonstrating the good performance of the LassoNet-RNN model, as indicated by the AUC value and F1-Score on the dataset of influencing people to develop depressive tendencies.

### 3.3 Importance of characteristics affecting depressive tendencies

This study was based on the LassoNet-RNN variable selection strategy to screen variables for important factors that influence a person's tendencies to depression and to generate importance rankings for the features. We first statistically analyzed the variables with depression and non-depression as shown in Table 5. And, the results of the 27 characteristic variables and variable importance obtained are shown in Table 6, where the variable importance for LassoNet-RNN is the maximum Lambda value recorded at the time the variable was screened out, this is because the Lambda value reflects the degree of contribution of the variable to the model. For example, the age feature was not dropped from the model until the Lasso regularization coefficient Lambda value exceeded 245.48, which indicates that as Lasso regularization coefficient Lambda values less than 245.48, the gain that the age feature brings to the model loss function is sufficient to compensate for the bias introduced by the regularization term corresponding to it, hence the coefficient of the age feature is not constrained to zero.

**Table 5:** Comparison of general characteristics of study participants with and without depression in final data.

Characteristics	Group	Depression	Non-depression	$\chi^{2}$	*p*
Gender	Male	384	1284	114.734	<0.001
	Female	871	1348		
Age	<50	24	45	2.676	0.262
	50–70	1016	2187		
	>70	215	400		
Residence	Rural	950	1840	14.056	<0.001
	Urban	305	792		
Education	<3	713	1096	102.915	<0.001
	≥3	542	1536		
Whether living with a spouse	Yes	982	2223	22.678	<0.001
	No	273	409		
Intergenerational financial support	Yes	1104	2229	7.479	<0.01
	No	151	403		
Availability of health insurance	Yes	1213	2564	1.799	0.180
	No	42	68		
Social participation	<3	636	1266	10.624	0.224
	3–7	374	793		
	>7	245	573		
Sleeping time at night	<6	625	722	187.777	<0.001
	≥6	630	1910		
Lunch break or not	Yes	743	1631	2.733	0.098
	No	512	1001		
Recently hospitalized	Yes	235	296	40.300	<0.001
	No	1020	2336		
Drinking frequency	0	947	1682	54.099	<0.001
	1	72	181		
	2	236	769		
Smoking or not	Yes	306	807	16.393	<0.001
	No	949	1825		
Presence of chronic diseases	Yes	713	1208	40.511	<0.001
	No	542	1424		
Availability of pensions	Yes	1131	2381	0.115	0.734
	No	124	251		
Whether working in agriculture	Yes	89	255	7.104	<0.01
	No	1166	2377		
Whether self-employed or privately owned	Yes	119	125	32.355	<0.001
	No	1136	2507		
Retirement	Yes	275	542	0.891	0.345
	No	980	2090		
Amount spent in a year	<8	415	926	1.682	0.195
	≥8	840	1706		
Whether owe a debt	Yes	60	107	1.058	0.304
	No	1195	2525		
Availability of piped water	Yes	961	2101	5.374	<0.05
	No	294	531		
Availability of bathing facilities	Yes	704	1726	32.604	<0.001
	No	551	906		
Availability of gas facilities	Yes	146	401	9.118	<0.01
	No	1109	2231		
Availability of heating	Yes	97	261	4.862	<0.05
	No	1158	2371		
Availability of broadband facilities	Yes	421	1157	38.210	<0.001
	No	834	1475		
Home tidiness	0–1	493	806	32.734	<0.001
	2	401	892		
	3–4	361	934		
Indoor temperature	0–1	37	54	4.333	0.363
	2	1034	2220		
	3–4	184	358

**Table 6:** Ranking the importance of characteristics affecting depressive tendencies.

Characteristic variables	Variable importance	Characteristic variables	Variable importance
Age	245.48	Recently hospitalized	95.52
Home tidiness	192.34	Lunch break or not	95.52
Education	192.34	Whether living with a spouse	88.11
Social participation	183.18	Intergenerational financial support	88.11
Drinking frequency	183.18	Smoking or not	88.11
Sleeping time at night	183.18	Whether working in agriculture	88.11
Indoor temperature	174.46	Whether self-employed or privately owned	83.92
Residence	174.46	Presence of chronic diseases	83.92
Gender	174.46	Availability of piped water	83.92
Availability of bathing facilities	174.46	Amount spent in a year	83.92
Availability of health insurance	158.24	Availability of heating	79.92
Availability of broadband facilities	150.70	Availability of gas facilities	76.11
Availability of pensions	112.46	Retirement	56.80
Whether owe a debt	112.46		

Note: This table lists the characteristic variables in descending order of importance.

**Fig. 7:**
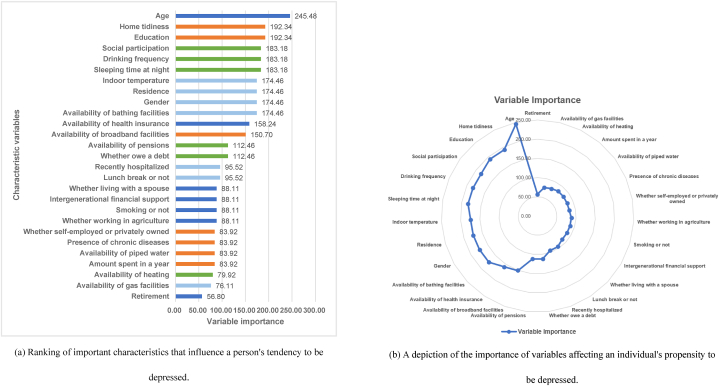
Important characteristics that influence a person's tendency to be depressed analysis.

The ranking of the Lambda values in Table 6 shows that of all the 27 influences on depression, the age of the individual is the first with a Lambda value of 245.48. The second two influences are home tidiness and education with a Lambda value of 192.34; social participation, drinking frequency, and sleeping time at night with a Lambda value of 183.18; followed by indoor temperature, place of residence, gender, and availability of bathing facilities with a Lambda value of 174.46; availability of health insurance with a Lambda value of 158.24; availability of broadband facilities was 150.70; availability of pensions and debt were both 112.46. The Lambda values for recent hospitalization and lunch break were 95.52; whether living with a spouse, intergenerational financial support, smoking, and agricultural work were 88.11. The remaining Lambda values were: self-employment (83.92), chronic illness (83.92), availability of piped water (83.92), amount spent in a year (83.92), availability of heating (79.92), availability of gas facilities (76.11), and retirement (56.80). Fig. 7 (a) illustrates the ranking chart of important factors that influence a person's tendency to become depressed. Fig. 7 (b) is a depiction of the importance of variables affecting an individual's propensity to be depressed.

## 4 Discussion

Most of the current research on the important influences that affect people's tendencies to depression have focused on the following areas. First, the physiological indicators and some sociological indicators have been singled out as important influences on depressive tendencies, with little indication of the degree of importance among these factors. Second, previous studies have often used cross-sectional data to explore the relationship between various factors and individual depression. Third, the impact on individual depression has focused more on large sample levels (such as morbidity and mortality) and have not assessed the influence of the individual factors on depressive tendencies. Although most of these studies have analyzed the relationships between depressive risk factors, they have largely been based on linear scientific models as the theoretical basis, limited to linear relationships, such as logistic models, which use simple and straightforward linear causality to explore the important factors that influence people's propensity to become depressed and, in doing so, to reveal deterministic patterns in people's various behaviors and to speculate on the evolution and transformation of such behavior in the future. However, the human psyche is open and complex, and simple linear models do not provide a good explanation of the influences that affect the development of depression in people and do not give a good indication of the importance of each factor. This has led to a focus on non-linear scientific concepts and methods in the study of depressive tendencies, which has provided a new perspective for understanding human psychology and behavior. Time series analysis is a very important statistical analysis method, widely used to analyze longitudinal data. It is also one of the most important tools for studying complex non-linear characteristics.

This study carried out an innovation in the network model by using the LassoNet-RNN model for feature selection on national follow-up survey data to identify the most important factors influencing a person's propensity to depression. Although the LassoNet model performs well on cross-sectional data, it is unable to capture features based on temporal relationships. And for some features, a time series representation is more likely to show the true nature of the features. While most current research on the important factors influencing people's depressive tendencies is at the linear stage, and time series are rarely used to identify important features. This study improves on the LassoNet feature selection model by combining the performance of RNNs on time series data, replacing the feedforward neural network of the hidden layer of LassoNet with an RNN, and constructing and generating a feature selection model LassoNet-RNN applicable to multivariate time series for classification or feature selection of time series with arbitrary time frequencies. LassoNet-RNN achieves feature sparsity by jumping layers and allows a feature to participate in an RNN layer only when the jump layer of that feature is active [58]. The model differs from other feature selection methods in that it uses a modified objective function with constraints, allowing feature selection to be combined directly with parameter learning. The model is therefore suitable for studying important features on datasets that affect people's propensity to depression. Therefore, in this study, the experiments of the algorithm are first based on several multivariate time series benchmark datasets, and the performance of the LassoNet-RNN algorithm is compared with the current major multivariate time series classification models, evaluated in terms of Accuracy. This is shown in Table 7. It can be seen that on some multivariate time series datasets, the accuracy of the LassoNet-RNN proposed in this study is higher than that of multivariate time series models such as Rockrt [74], IT [49], Weasel-Muse [51], CIF [75], HC and ResNet [76].

**Table 7:** Experiment results of LassoNet-RNN model and other models.

	Rockrt	IT	TSF	CIF	Weasel-Muse	STC	HC	ResNet	DTW_A_	TapNet	LassoNet-RNN
AF	0.249	0.220	0.298	0.251	0.740	0.318	0.293	0.369	0.224	0.302	0.740
BM	0.990	1.000	0.998	0.983	0.997	0.979	1.000	1.000	0.990	0.991	0.990
ER	0.981	0.921	0.898	0.957	0.969	0.843	0.943	0.872	0.929	0.895	1.000
SRS1	0.866	0.847	0.847	0.860	0.736	0.847	0.860	0.761	0.813	0.813	0.932
SRS2	0.513	0.521	0.507	0.489	0.495	0.516	0.517	0.502	0.524	0.535	0.600
SWJ	0.456	0.420	0.333	0.451	0.347	0.440	0.401	0.309	0.256	0.351	0.733
UW	0.944	0.912	0.851	0.924	0.904	0.870	0.913	0.884	0.915	0.884	0.958
HMD	0.446	0.426	0.485	0.522	0.380	0.349	0.378	0.353	0.307	0.323	0.608

Note: The values compared are all accuracy.

In addition, this study used the proposed LassoNet-RNN algorithm for feature selection affecting depressive tendencies to obtain a ranking of the importance of influencing factors that affect people's psychological tendency to become depressed. In this study, a large amount of data from middle-aged and elderly individuals is used as a sample, and based on the LassoNet-RNN method, the data is trained and modeled to find out the important factors affecting people's depressive tendencies.

Considering the characteristics of the important factors that influence people's propensity to depression, the pathogenesis of depression is complex and influenced by a variety of factors such as demographics, health-related risk factors, family economic status, and residential environment [59,64,66,69,70]. Exploring the risk factors that contribute to the development of depressive psychology has positive implications for people's mental health. By analyzing and mining the CHARLS dataset, a screening model of factors influencing depressive tendencies was constructed for researchers in public health. The specific findings are as follows:

In terms of socio-demographic data, for example, depression is associated with factors such as education, intergenerational support, whether or not one lives with one's spouse and where one lives [77]. Higher education often means higher income and status, which leads to more resources, including access to health care. Education enables individuals to have better access to information and improved critical thinking skills, which can lead to improved health behaviors. It is also important to emphasize the importance of family, colleagues and peers who can provide emotional and practical support. When provided with high-quality support, individuals feel more optimistic and are able to mobilize their resources more quickly when faced with a threat, thereby reducing the incidence of depression. In terms of health-related risk factors, such as drinking alcohol and sleep can influence the occurrence of depression. In turn, depressive mental states can cause or exacerbate sleep disorders in people. Depression can be reduced by taking steps to improve physical fitness and increase social interaction to help people achieve physical health to maintain a good mental state. In terms of economic status, the importance of the various influencing factors varies little. Other influencing factors (whether one is engaged in agricultural activities and whether one is self-employed) are significantly associated with depressive tendencies. In terms of living environment, the home tidiness is the most important factor influencing a person's tendency to become depressed. And, the availability of broadband is associated with depression, as broadband internet communication providing a basic facility for contact with the outside world, relieving loneliness and helping to improve people's subjective sense of well-being. Adequate electricity, running water, gas, and heating are also associated with individual depression. These services matter because they could improve the indoor environments in terms of light, humidity, and temperature, further contributing to people's physical comfort and psychological well-being.

In addition to objective disease triggers such as genetics, geographical location, and natural conditions, the specific environments in which people grow up, live, study and work, as well as the broader factors that influence these environments and the diverse population groups, cannot be ignored as the influencing psychological impact of depression [78,79]. Scientific social interventions can therefore help to reduce the probability of illness, such as strengthening universal mental health screening. The aim of this study is to support interdisciplinary pathogenic research to identify the social and biological co-determinants of depression in time and space, and to call on all relevant groups in society to work together to achieve the ultimate goal of mental health well-being and spiritual well-being for all groups.

## 5 Limitations and future works

In this study, LassoNet is combined with RNNs to construct feature selection models. The LassoNet-RNN multivariate time series model was constructed by replacing the feedforward neural network in the hidden layer of the LassoNet model with an RNN that can identify time series features, in combination with the performance of RNN on time series data. The LassoNet-RNN model is used to construct a screening model for factors influencing depressive tendencies on the CHARLS dataset, to analyze and mine the data for important factors influencing people's tendencies to become depressed, to obtain a ranking of the importance of the characteristic variables, and to synthesize the relevant results and provide recommendations for decision-makers and researchers in the field of public health. However, there are some limitations and shortcomings in this study, which are listed below and will be addressed in future work and research.

First, since the hidden layer of the LassoNet network structure is a feed-forward neural network. This hidden layer structure restricts the LassoNet model to cross-sectional data only. To capture the continuous features that influencing factors of depression, a neural network architecture that identifies multivariate time series is required. To enhance the LassoNet model, this study utilized the RNN [45] in replacing the hidden layer. Other RNN types and variants, such as LSTM, bidirectional RNNs, multidimensional RNNs, and other RNN network structures, are not explored in this study. How well the other types of RNN networks work in conjunction with the LassoNet model is a direction that could be investigated next. Many of the machine learning studies included in this study suffer from a low sample size and low dimensionality. For example, the CHARLS dataset has less than 10,000 samples after removing missing values, and the number of features is not large enough. These limitations increase the possibility of overfitting, and the efficacy of LassoNet-RNN models on larger multivariate time series datasets should be investigated by researchers in the future.

Second, the research data in this study is somewhat limited and the selection of factors influencing depressive tendencies is not comprehensive and lacks a practical basis. In order to more accurately identify the factors that influence people's tendencies to depression, the next step should involve selecting the characteristic variables based on the specific practical situation. For example, the CHARLS dataset used in this study only includes middle-aged and older adults over 45 years of age. Due to the limitations of this dataset, our selection of factors influencing depressive tendencies is limited, which may lead to biased results. Future research should consider using datasets that include individuals of all age groups to identify crucial factors that influence depressive tendencies.

Finally, our study identifies the importance of factors influencing individual depressive tendencies based on the CHARLS dataset. The initiatives and detailed recommendations given for the important influences associated with depressive tendencies obtained are not specific, in-depth, and lack more detailed methodological steps. Future research could conduct more in-depth analyses to provide specific and detailed recommendations, as well as more meaningful approaches and methodologies.

## Funding

This study was funded by 10.13039/501100001809National Natural Science Foundation of China (72271126, 12227808), Major Project of Natural Science Foundation of Jiangsu Education Department (22KJA630001), Postgraduate Research and Practice Innovation Program of 10.13039/501100002949Jiangsu Province (KYCX23_2343), College Student Innovation and Entrepreneurship Training Program (202211287083Y), and Qinglan Project of 10.13039/501100002949Jiangsu Province.

## Data availability statement

Data will be made available on request.

## CRediT authorship contribution statement

**Jiatong Han:** Conceptualization, Investigation, Writing – original draft, Writing – review & editing. **Hao Li:** Conceptualization, Investigation, Writing – original draft, Writing – review & editing. **Han Lin:** Conceptualization, Investigation, Writing – original draft, Writing – review & editing. **Pingping Wu:** Investigation, Methodology, Writing – original draft, Writing – review & editing. **Shidan Wang:** Investigation, Methodology, Writing – original draft. **Juan Tu:** Methodology, Resources, Software, Writing – review & editing. **Jing Lu:** Methodology, Resources, Software, Writing – review & editing.

## Declaration of competing interest

The authors declare that they have no known competing financial interests or personal relationships that could have appeared to influence the work reported in this paper.
